# Knowledge and Perceptions of Parents and Adolescents Regarding HPV and Its Vaccine

**DOI:** 10.7759/cureus.102411

**Published:** 2026-01-27

**Authors:** Eleni Tsimpou, Anastasia Bothou, Christina I Nanou, Giannoula A Kyrkou, Pinelopi Varela, Athina Diamanti, Victoria Vivilaki, Anna Deltsidou

**Affiliations:** 1 Department of Midwifery, Faculty of Health and Care Sciences, University of West Attica, Athens, GRC

**Keywords:** hpv clinical manifestations, hpv infection, hpv transmission, hpv vaccine, human papilloma virus (hpv)

## Abstract

Background

Human papillomavirus (HPV) is one of the most common sexually transmitted infections worldwide and is associated with several malignancies. Understanding parental and adolescent knowledge and perceptions regarding HPV and its vaccine is essential for improving vaccination uptake.

Methods

This cross-sectional study was conducted between April and May 2023 in a health center in Northern Greece. Questionnaires were completed by 151 parents and 152 adolescents aged 11-18 years. Data were analyzed using descriptive statistics and multivariable regression analyses.

Results

Among parents, 89.3% were aware of HPV, and 82.8% were generally in favor of vaccination. Higher HPV knowledge scores were observed among women and participants with higher educational levels (p < 0.001 and p = 0.001, respectively). Among adolescents, 63.6% expressed intention to receive the HPV vaccine. Female gender (odds ratio (OR) = 2.16, 95% confidence interval (CI): 1.05-4.46) and discussion with parents about sexually transmitted diseases (STDs) were independently associated with vaccination intention. Older age was positively associated with higher HPV knowledge scores among adolescents (p < 0.001).

Conclusions

Both parents and adolescents demonstrated substantial awareness of HPV infection and its prevention. Parental communication and adolescents’ gender were important factors associated with vaccination intention, highlighting the need for targeted educational interventions to enhance HPV vaccine uptake.

## Introduction

Human papillomavirus (HPV) infection is one of the most important and complex infections and is of great interest as it is the most common sexually transmitted disease (STD). It has been estimated that three out of four sexually active individuals (men and women) will be infected with some subtype of HPV in their lifetime, and most of them will remain asymptomatic or self-infected after one to two years [1]. It is an infection that can cause warts and malignant neoplasms in the cervix, penis, anus, nasopharynx, and tongue [2-3]. The potential to cause cancer depends mainly on the subtype of HPV.

About 200 HPV subtypes have been described, but 15 of them account for 99.7% of cervical cancer and are classified as high-risk HPV subtypes [4-5].

The virus is spread by simple touch with infected skin, as well as sexual interaction between the skin and epithelium, while there is currently no evidence of blood-borne transmission. Because of the high risk of infection and the increased tendency of specific subtypes to cause cancer, vaccines have been developed as a primary defense against the virus.

Because it lowers the risk of cancer, the HPV vaccine is regarded as one of the most significant individual and societal initiatives. There are currently three vaccinations available: 1) the quadrivalent Gardasil, approved for distribution in 2006; 2) the twin vaccine Cervarix, which was licensed in 2007 [6]; and 3) the nine-valent vaccine known as Gardasil-9, which was finally approved in 2014 [7]. The vaccinations mentioned above are all meant to prevent rather than treat, and they work best when administered early in life, before the initiation of sexual activity.

The HPV vaccine is characterized by high efficacy and safety; however, research data indicate that the population is unaware of both HPV and the HPV vaccine. Contributing to this is the lack of information on STDs from both the school environment and the family.

Despite the global burden of HPV infection and the well-established effectiveness of HPV vaccination programs, vaccination uptake remains suboptimal in several countries, including Greece. Parental beliefs, adolescents’ perceptions, and communication within the family have been identified as key determinants of vaccination behavior. However, limited data are available regarding how global HPV-related public health concerns translate into local knowledge, attitudes, and vaccination intentions among parents and adolescents in the Greek setting.

The primary aim of this cross-sectional study was to assess the level of knowledge and perceptions of parents and adolescents regarding HPV infection and HPV vaccination. A secondary aim was to identify factors associated with adolescents’ and parents’ intentions toward HPV vaccination.

## Materials and methods

Design of the study

The study was conducted at one health center in Northern Greece from April 1, 2023, to May 31, 2023, after approval from the Ethics Committee of the University of West Attica (protocol code: 52242/30-05-2023) and by the Scientific Council of the 3rd Health District, where the health center belongs (protocol code: 14166/16-05-2023). The study population consisted of adolescents aged 12 to 18 and their parents who came to the pediatric clinic of the health center either for a scheduled appointment or for an emergency visit due to illness. The criteria for parental participation in the study were to be parents of children aged 12 to 18 years, with no distinction in the gender of the parents or the gender of their child. Participants were recruited using a convenience sampling approach, consisting of consecutive adolescents and their parents attending the pediatric clinic of the health center during the study period. 

The questionnaires consisted of closed-ended items assessing knowledge about HPV infection (transmission routes, health consequences, and prevention), attitudes toward HPV vaccination, and vaccination intention, which were freely available online and for which no permission was required to use them (see Appendix) [8]. They were translated from English into Greek by two bilingual persons and were designed to collect information about their knowledge of HPV infection and their attitudes toward vaccination. Internal consistency was assessed in the present sample, demonstrating acceptable reliability (Cronbach’s α > 0.7). Both study groups completed the questionnaires anonymously, which were accompanied by written informed consent describing the purpose of the study. Written parental consent was obtained for participating adolescents under 15 years of age.

Statistical analysis

Using the Kolmogorov-Smirnov criterion, the distributions of the quantitative variables were tested for normality of their distribution. For those that were normally distributed, the means and standard deviations (SD) were used to describe them, while for those that were not normally distributed, the median and the interquartile range were also used. Absolute (N) and relative (%) frequencies were used to describe the qualitative variables. The internal reliability of the questionnaires was tested using Cronbach's alpha coefficient. Spearman's correlation coefficient (rho) was used to test the relationship between two quantitative variables. The non-parametric Mann-Whitney criterion was used to compare quantitative variables between two groups. For the comparison of quantitative variables between more than two groups, the non-parametric Kruskal-Wallis criterion was used. To control for type I error due to multiple comparisons, the Bonferroni correction was used, according to which the significance level is 0.05/k (k = number of comparisons).

Variables considered for inclusion in the multivariable regression models included demographic characteristics (age, gender, educational level), HPV-related knowledge scores, attitudes toward vaccination, sexual behavior variables (where applicable), and communication with parents regarding STDs. Variables that showed statistical significance in univariable analyses or were considered clinically relevant based on the literature were entered into the multivariable models using a stepwise selection approach.

To identify independent factors linked to HPV knowledge, a stepwise inclusion/exclusion approach in linear regression analysis was employed. From these components, dependency coefficients (b) and their standard errors (SE) were derived. When the dependent's distribution was non-normal, logarithmic modifications were used to do a linear regression analysis. To identify independent variables connected to vaccination intention, logistic regression analysis was used. Odds ratios and their 95% confidence intervals (95% CI) were produced. The statistical significance threshold was set at 0.05, and the significance levels are two-sided. The analysis was conducted using the IBM SPSS Statistics for Windows, version 26.0 (released 2012, IBM Corp., Armonk, NY).

## Results

Results on the sample of parents

The sample consisted of 151 parents with a mean age of 44.2 years (SD = 5.3 years). One-hundred thirty-one (86.8%) parents were women. Sixty-nine (45%) were high school graduates, and 24 (15.9%) were office workers. The majority (97; 85.1%) had full-time employment. Moreover, 87 (57.6%) had two children. The median age for the first child attending the center was 14.5 years (range 13-16 years), for the second 12 years (range 12-14 years), and for the third 12 years (range 10-13 years). Finally, 56 (37.3%) had a family history of cancer, and 19 (12.8%) had a history of uterine cancer (Table 1).

**Table 1:** Demographic characteristics of the parents

		Ν	%
Age, mean (SD), median (range)		44.2 (5.3)	44.0 (42 – 48)
Gender	Male	20	13.2
	Female	131	86.8
Educational level	Junior high school graduate	23	15.2
	High school graduate	69	45.7
	University graduate	45	29.8
	Master's degree	9	6.0
	Doctorate (PhD)	5	3.3
Profession	Unemployed	21	13.9
	Household	23	15.2
	Employee	16	10.6
	Office worker	24	15,9
	Manager	4	2.6
	Self-employed	20	13.2
	Other	43	28.5
Professional employment	Full-time	97	85.1
	Part-time	17	14.9
Number of children	1	29	19.2
	2	87	57.6
	3	30	19.9
	4	5	3.3

One-hundred twenty-five (82.8%) were generally in favor of vaccination. Among those who were against vaccinations, 19 (50%) thought that vaccinations might cause problems. On the other hand, among those who were in favor, 101 (67.2%) vaccinated their children because of the pediatrician’s recommendation. Finally, 114 (75.5%) of the participants' children's vaccination was significantly influenced by the pediatrician.

One hundred thirty-five (89.3%) knew about HPV, and 117 (77.5%) thought it could be dangerous. One hundred seventeen (77.5%) knew that it is transmitted through sexual intercourse, and 95 (62.9%) thought it could affect their children. However, among those who did not believe it, 19/38 (50%) were in favor of this view because their children had not yet had sexual intercourse, and 34 (22.2%) because they had no family history of cancer. Finally, 112 (74.2%) knew that the main aim of vaccination is to prevent cervical cancer, and 43 (28.5%) knew that it also prevents STDs.

One hundred fourteen (75.5%) wanted their children to be vaccinated, 110 (72.8%) wanted to be vaccinated to prevent a potentially cancerous infection, and 73 (48.3%) thought it should be given at the age of 12-13 years. By contrast, 125 (82.6%) of those who did not want to be vaccinated were because they were afraid of the side effects. Also, 117 (77.2%) of the sample believed that vaccination for HPV is recommended before starting sexual activity, and 141 (93.4%) wanted more information about the virus and its prevention. Two (20%) of those who did not want more information believed that the virus does not concern them and their children, while 91 (60.3%) of those who wished to seek more information would seek it in private discussions with doctors of different specialties. Finally, 63 (41.6%) considered 12-13 years of age to be an appropriate age to start receiving information about the virus.

Seventy-nine (52.3%) of parents said they always discuss sexual issues with their children, and 87 (57.6%) said they always discuss STDs. In addition, 139 (92.1%) believed that children should be informed about HPV and its prevention. Ninety-nine percent of those who did not believe in the information believed that parents should decide for their children, while 112 (74.1%) of those who believed in the information wanted it to be given by a pediatrician or family doctor.

**Figure 1:**
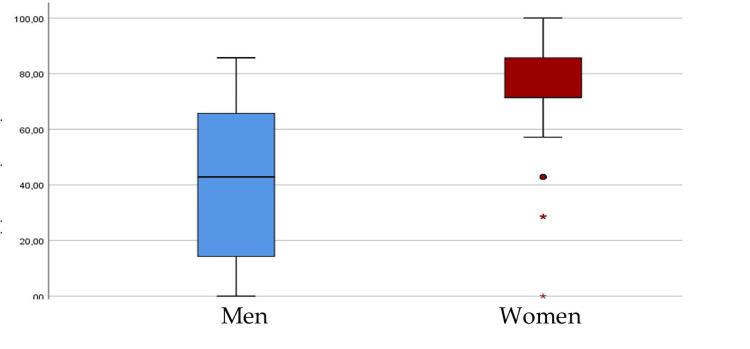
Parents' HPV knowledge scores by gender Image created by the authors with Microsoft Word (Microsoft Office Suite, Microsoft Corp., USA)

A knowledge score was then created from the questions for which the correct answer was marked. Each correct answer was given a value of 1, and each incorrect answer was given a value of 0. The score was calculated and converted to a % score. Higher scores implied more knowledge about HPV. For this sample, the minimum value of the score, which was scored by four people (2.6%), was 0, and the maximum, which was scored by 18 people (11.9%), was 100, while the mean value was 70.6 points (SD = 23.7 points). The Cronbach's reliability coefficient a was greater than 0.7, indicating acceptable reliability. Knowledge scores were found to differ according to gender, educational level, family history of cancer, specifically uterine cancer, and positive or negative attitudes toward vaccination. In particular, women had significantly more knowledge compared to men (p < 0.001) (Figure 1).

After Bonferroni's correction, university graduates demonstrated greater knowledge than high school graduates (p < 0.008). Similarly, pro-vaccination individuals generally possessed more knowledge about the virus than anti-vaccination individuals (p < 0.001).

Parents' age was not significantly associated with their knowledge of HPV. Those who believed the virus could affect their children had more knowledge about it than those who did not (p < 0.001). Similarly, those who wanted their children to be vaccinated had more knowledge compared to those who did not want to or were not sure (p < 0.001). Finally, after Bonferroni's correction, those who always discussed sexual matters with their children had significantly higher scores compared to those who never discussed them (p = 0.02).

After a multivariate linear regression, educational level, gender, the general attitude towards vaccination, and belief that the virus can affect the participants' children were found to be independently associated with HPV knowledge score (Table 2).

**Table 2:** Multivariate linear regression with the dependent variable (knowledge score) and independent variables (participants' demographics, their views and attitudes towards vaccination and the virus, and the extent of discussion with their children about sex-related issues) The logarithm of the dependent variable has been used.

Parameter	β+	SE++	b÷	P
Educational level				
Secondary school graduate versus high school graduate	0.050	0.068	0.073	0.459
University graduate versus secondary school graduate	0.185	0.071	0.248	0.010
Postgraduate/doctoral degree (PhD) versus secondary school graduate	0.231	0.093	0.196	0.014
Gender (female versus male)	0.386	0.072	0.383	<0.001
Are you in favor of vaccinations in general? (Yes vs. No)	0.156	0.066	0.172	0.020
Do you think HPV can affect your children? (Yes vs. No)	0.162	0.050	0.230	0.002

Based on the results, a) parents who were university graduates or held a master's/doctoral degree had more knowledge than high school graduates, b) women knew more about the virus compared to men, c) those who were pro-vaccination had more knowledge about vaccination than those who were not, and d) those who believed the virus could affect their children had more knowledge about it compared to those who did not.

After a multivariate linear regression, general attitudes towards vaccination, the belief that their children might be infected with the virus, and discussion of STDs were found to be independently associated with vaccinating their children for HPV (Table 3).

**Table 3:** Multivariate logistic regression with the dependent variable (the intention to vaccinate children) and independent variables (the participants' demographic data, their general attitude towards vaccinations and the virus, and the degree of discussion with children about sexual issues) Ratio (95% confidence interval)

	OR (95% CI)+	P
Do you discuss STDs with your parents?		
Only if they bring it up versus never	19.66 (2.63–146.91)	0.004
Only if I have a specific problem vs Never	1.32 (0.18–9.80)	0.783
Always versus never	2.87 (0.68–12.12)	0.151
Are you in favor of vaccinations in general? (Yes vs. No)	26.37 (6.70–103.86)	<0.001
Do you think HPV can affect your children? (Yes vs. No)	4.84 (1.71–13.74)	0.003

Based on the results, a) those who are generally pro-vaccination are 26.37 times more likely to want to vaccinate their children for this virus as well, compared to those who are not generally pro-vaccination; b) those who believed that the virus could affect their children were 4.84 times more likely to want to vaccinate them compared to those who did not; c) and those who discussed STDs with their children when they asked were 19.66 times more likely to want to vaccinate them compared to those who never discussed it.

Results on the adolescent participants

The sample consisted of 152 adolescents with a mean age of 14.5 years (SD = 1.8 years). Eighty-two (53.9%) of them were girls, and 70 (46.1%) were boys. Eighty-four (55.3%) adolescents had heard of HPV, and 85 (55.9%) considered it dangerous. Moreover, 79 (52%) knew that it is transmitted through sexual intercourse, and 89 (58.3%) were aware of the vaccine. Among them, 103 (67.8%) knew that the aim was to prevent cervical cancer, and 26 (17.2%) to prevent STDs. Forty-four (28.7%) had heard about the vaccine from their parents, 38 (24.7%) from their pediatrician, and 19 (12.7%) from teachers.

Eighty-nine (58.6%) of the sample thought they were affected by HPV, and 97 (63.6%) wanted to get the vaccine. Among those who did not want it, 28 (50%) said the reason was that they did not know where it was targeted. Similarly, among those who wanted to, 79 (81.2%) said the reason was to prevent cancer. Finally, 76 (50%) thought that vaccination should be carried out before sexual activity.

Eighty-three (54.6%) correctly considered that information about the possibility of HPV prevention should be provided at the age of 10-13 years. One hundred six (69.5%) wanted additional information about HPV and its prevention. Among those who did not need additional information, 6 (13%) believed it was because they had not had sexual intercourse or because they used a condom. However, among those who needed more information, 72 (68%) would like to receive it from a pediatrician.

Fifty-six (36.8%) of the participating adolescents had a boy/girlfriend, and 54 (35.8%) had at least one sexual intercourse on average at the age of 15 years (SD = 1.1 years). Among those who had sexual intercourse (n = 54), 31 (56.6%) had sexual intercourse with one person, and 36 (66.7%) used contraceptive methods. Among those who used contraceptive methods (n = 36), 31 (85.7%) reported condoms as the main method. Also, adolescents discussed their sexual issues mainly with friends 104 (68.4%) and with parents (53; 34.9%). Forty-six (30.3%) discussed sexual matters with parents only if they initiated the discussion, while 54 (35.5%) never discussed STDs with their parents.

A knowledge score was then created from the questions for which the correct answer was marked. Each correct answer was given a value of 1, and each incorrect answer was given a value of 0. The score was calculated and converted to a % score. Higher scores implied more knowledge about HPV. For this sample, the minimum value of the score that 16 people (10.5%) scored was 0, and the maximum, which 15 people (9.9%) scored 100, while the mean value was 54 points (SD=31 points). Cronbach's alpha reliability coefficient was greater than 0.7, indicating acceptable reliability.

**Figure 2:**
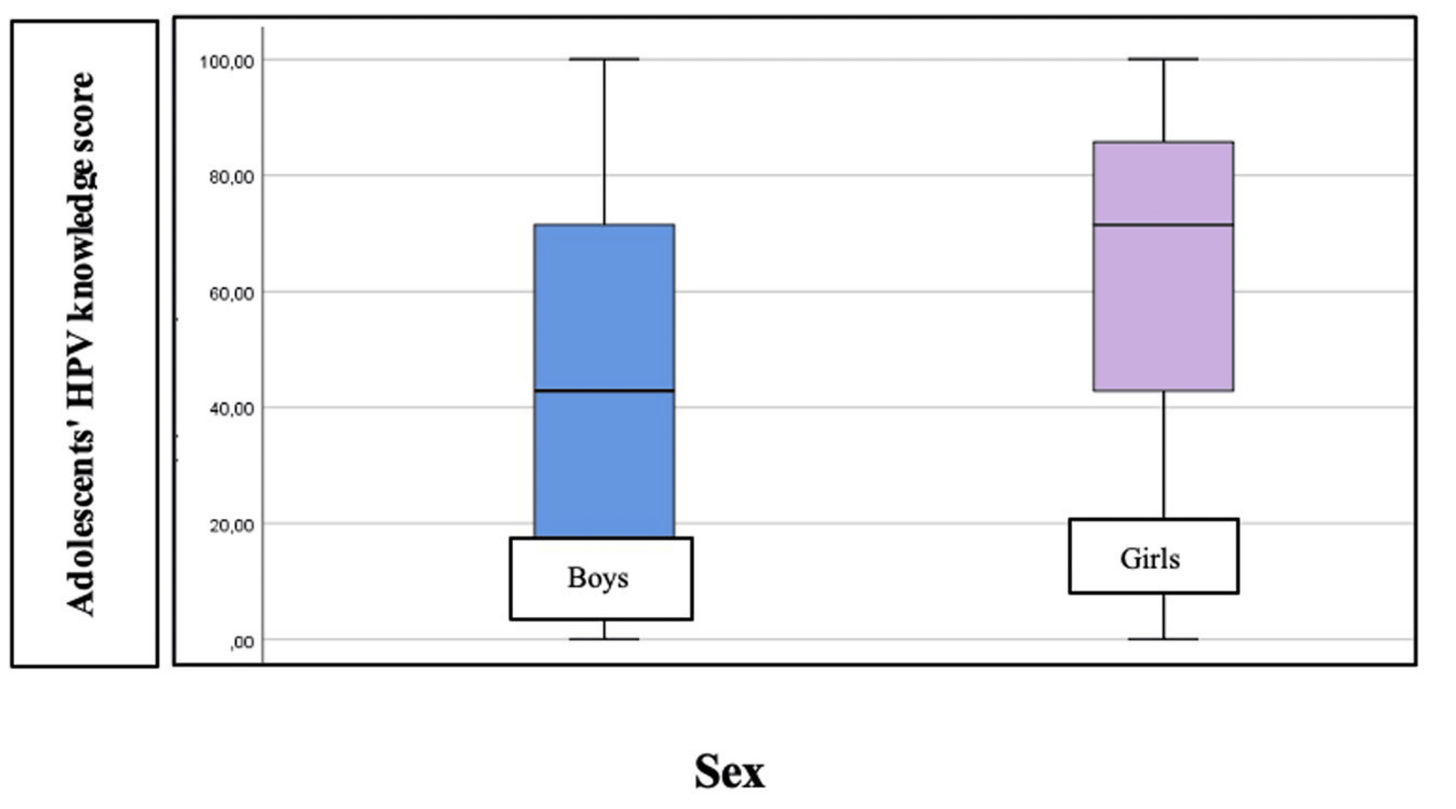
Adolescents' knowledge scores by gender Image created by the authors with Microsoft Word (Microsoft Office Suite, Microsoft Corp., USA)

The knowledge score was found to differ by gender, with girls having significantly more knowledge about the virus than boys (p = 0.001) (Figure 2).

**Figure 3:**
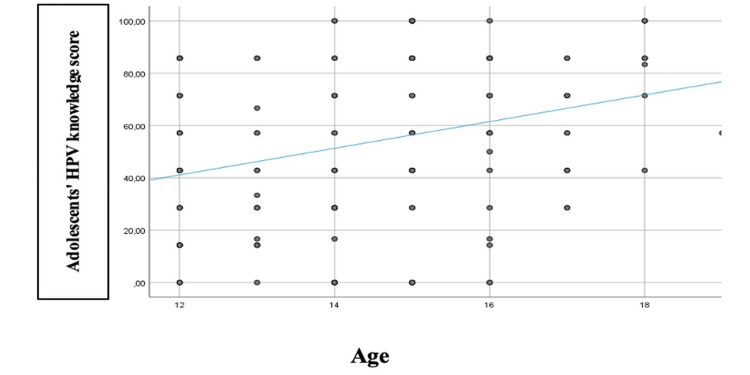
Correlation of adolescents' age with their HPV knowledge score Image created by the authors with Microsoft Word (Microsoft Office Suite, Microsoft Corp., USA)

Those who had been informed by a doctor other than a pediatrician (p = 0.012), parents (p = 0.008), or friends (p = 0.049) had more knowledge than those who had not informed. In addition, vaccination intention was associated with higher knowledge scores (p < 0.001) and sexual intercourse. Those who had at least one sexual intercourse had more knowledge about the virus compared to those who had no intercourse (p = 0.015). Still, after Bonferroni's correction, it was found that those who always discussed sexual matters with their parents compared to those who never discussed STDs compared to those who always discussed or even only when parents discussed or when they had a problem were more knowledgeable (p = 0.007 and p < 0.001, respectively). Adolescent age was found to be significantly and positively associated with HPV knowledge scores (Figure 3). Older age implied more knowledge (p < 0.001).

After a multivariate linear regression, intention to vaccinate and talking to parents about STDs were found to be independently associated with HPV knowledge score (Table 4).

**Table 4:** Multivariate linear regression with the dependent variable (knowledge score) and the independent variables (the demographics of the participating adolescents, their opinions and knowledge about vaccination, their sexual activity and use of precautions, and the degree of discussion with their parents about sexual issues) Dependence coefficient ++standard error ÷ standardized coefficient. The logarithm of the dependent has been used.

Parameter	β+	SE++	b÷	P	
Intention to vaccinate (Yes vs. No)	0.661	0.080	0.548	<0.001	
Talking about STDs with your parents?					
Only if they initiate it vs. Never	0.147	0.095	0.115	0.126	
Only if I have a specific problem vs Never	0.141	0.113	0.089	0.215	
Always vs Never	0.341	0.107	0.235	0.002	

Specifically, adolescents who had the intention to vaccinate knew more about the virus compared to those who did not, and adolescents who always discussed STDs with their parents had more knowledge compared to those who never discussed them.

Moreover, after a multivariate logistic regression, adolescents' gender and discussion with parents about STDs were found to be independently associated with HPV vaccination intention (Table 5).

**Table 5:** Multivariate logistic regression with the dependent variable (adolescents' intention to vaccinate) and independent variables (the participants' demographics, opinions and information about vaccination, their sexual activity and use of precautions, and the degree of discussion with parents about sexual issues) +Ratio (95% confidence interval)

Parameter	OR (95% CI)+	P
Do you discuss STDs with your parents?		
Only if they initiate it vs. Never	2.52 (1.06–6.02)	0.037
Only if I have a specific problem vs. Never	1.34 (0.48–3.74)	0.580
Always vs. Never	2.56 (0.90–7.28)	0.079
Gender (Female vs. Male)	2.16 (1.05–4.46)	0.037

Specifically, girls were 2.16 times more likely to want to be vaccinated compared to boys, and adolescents who always discussed STDs with their parents were 2.56 times more likely to want to get vaccinated compared to those who never discussed it.

## Discussion

In recent decades, the vaccination program against HPV has been expanded to reach as many age groups as possible, while recently an attempt has been made to immunize adolescent boys. Despite the widespread public awareness of the undoubted adverse health effects of the virus and the value of protection through vaccination, there are still instances of negative attitudes, hesitation, and skepticism about the benefits of the HPV vaccine [9].

Based on the results of our study, it was found that the majority of the parents were informed about HPV and recognized its potential risks. Similarly, parents demonstrated awareness of HPV transmission and its relevance to their children’s health. These findings are in line with the results of the study by Grandahl et al. (2018), according to which parents' knowledge levels are particularly high, which contributes to their positive attitude towards vaccinating their children [10].

Among parents who did not consider this infection as likely to affect their children, the majority of them based their opinion on the certainty that their children were not sexually active, while an equally large proportion considered that, given the absence of a family history of cancer, there was no reason for concern about infection with this virus. This conclusion is also reached in the study by Rancic et al. (2022) [11].

Parents' attitudes regarding the vaccination of their children against HPV were largely positive, although, at the same time, they raised some concerns about the overall vaccination process. These results are also concluded by other studies [12-13], in which parents' hesitancy about HPV vaccination was associated with their general negative attitude towards vaccination and its safety.

In our study, many parents also indicated a need for additional information on this topic, demonstrating their rising anxiety and desire to learn more about HPV and how to protect their children from it. This is similar to a study conducted in 2022 by Rancic et al. [11]. Many parents believed that HPV vaccination should occur before sexual debut. Concurrently, parents consulted medical professionals to obtain a thorough knowledge of the virus and obtain an HPV vaccination, according to a 2018 study by Lee et al. [14].

Although the highest percentage of participants want their children to be vaccinated against HPV, only half of them believe that this vaccination should be carried out at an early age, around 12 to 13 years. Despite any reservations they may have, a significant proportion of the parents who took part in the study consider 12 to 13 years of age to be an appropriate age for informing their children about this virus, its risks, and ways of preventing its transmission. The study by Seven et al. (2015) [15] also finds the tendency of parents to carry out vaccination of their children at around 13 years of age, with the study by Rancic et al. (2022) [11] reaching similar results.

The level of parental information is found to vary significantly according to gender, educational level, family history of cancer, specifically uterine cancer, as well as generally positive or negative attitudes towards vaccination. In particular, among the parents, women appear to be more informed than male participants, which is not surprising given that this is a virus that causes significant cervical lesions. Increased levels of awareness among women were also recorded in the study by Ogochukwu et al. (2017) [16].

Regarding the correlation between the participants' educational level and their attitude towards HPV vaccination, higher education graduates appear to be more informed than secondary school graduates. Similarly, a significant correlation is found between parental attitudes towards vaccination in general and their awareness of infection and HPV vaccination. No associations were found between the annual income of the participating parents and their levels of information about HPV vaccination. It is also important that a significant number of parents report that they openly discuss sexual issues and issues related to STDs with their children, which contributes to optimal information for children about the potential risk of HPV infection. Similar results were also found in other studies [11-12].

At the same time, a remarkably high percentage of participating parents seem optimistic about informing their children about HPV and ways to prevent infection. Parents' information about their children regarding the existing risks of HPV infection is also supported by the study by Shato et al. (2023) [13], with parents considering informing their children as a primary factor in preventing infection.

Adolescents appeared to be aware of the infection and its risk, of the transmission of the virus through sexual contact, and of the benefits of HPV vaccination, and in particular its contribution to the prevention of cervical cancer. The source of information for most adolescents was their parents, with equally important information from their teachers and their pediatrician. The study by Carvalho and Araújo (2021) [17] also found a correlation between adolescents' awareness of the risk of infection, their awareness of the benefits of the vaccine, and their intention to receive information on this topic from their parents.

It is important to note that most adolescents want to be vaccinated and consider that their vaccination should be carried out before they start sexual activity. Some of them state that information about the possibility of HPV prevention should be provided at a young age, around 10 to 13 years old, with their pediatrician as the most appropriate source of information. The correlation between the age of adolescents and their levels of information regarding this infection was also identified in the study by Santos et al. (2020) [18]. Similarly, the survey by Icardi et al. (2020) [19] concluded that adolescents' attitudes towards vaccination are shaped by their levels of information, with the internet and friends being their primary sources of information.

A small proportion of the sample reported being sexually active, with an average age of onset of 15 years. Most of them reported having a single partner and using contraceptive methods, with condoms being the primary contraceptive method. A high percentage of the participating adolescents say they are comfortable discussing sexual issues with their friends, while a small percentage discuss such issues with their parents on their own initiative. Similar results regarding adolescents' reduced levels of comfort in discussing topics of a sexual nature with their parents were found in the study by González-Cano et al. (2021) [20].

In terms of socio-demographic characteristics of the participating adolescents, girls show higher levels of awareness of the virus. Adolescents who reported being sexually active appear to be more informed than adolescents who were not sexually active. Similarly, high levels of HPV knowledge are shown by adolescents who discuss sexual matters with their parents, compared to those who avoid such discussions. The age of adolescents was found to be positively significantly correlated with their level of HPV awareness, with older people being more knowledgeable. Similar results were also found in the study by Junior et al. (2023) [21], where a clear difference was also observed between the levels of knowledge and awareness of girls versus boys on HPV.

Overall, these findings suggest that parental education and gender are key determinants of HPV-related knowledge, while family communication plays a central role in shaping adolescents’ intention toward HPV vaccination.

The intention to vaccinate against HPV is increased among adolescents who are aware of the virus, but also among those who have an active sexual life. There is also a positive correlation between vaccination intention and discussion with parents about STDs. There is also a positive association between gender and discussion with parents about STDs, with girls showing higher rates of engagement in this type of discussion than boys. Similarly, in the study by Oliveira et al. (2020) [22], it was found that adolescents who were not sufficiently informed about the risk of the disease remained unvaccinated, a point indicating the association between adolescents' awareness and their vaccination levels against HPV.

In regions with low HPV vaccination coverage, from a public-health perspective, the findings underscore the importance of school-based educational interventions that provide age-appropriate information on HPV and its prevention. In the Greek context, structured collaboration between schools, healthcare professionals, and families could enhance adolescents’ knowledge and support informed decision-making regarding HPV vaccination.

Limitations

This study has certain limitations that should be acknowledged. The study was conducted in a single health center in Greece, which may limit the generalizability of the findings to broader populations. Moreover, although the sample size was adequate for statistical analysis, it was relatively modest and may not fully capture the diversity of parental and adolescent perspectives. Finally, as the study relied on self-reported questionnaires, some recall or social desirability bias cannot be excluded. Despite these limitations, the study offers valuable insights into the knowledge and attitudes of parents and adolescents regarding HPV and vaccination, highlighting important directions for health education and future research.

## Conclusions

HPV was known to most parents before they participated in the study. Parents' attitudes toward vaccination are based on their awareness of the risk of the disease and how to prevent it, and appear to be positive, with a relatively high percentage of parents wanting to vaccinate their children. Based on adolescents' results, a clear correlation was found between their levels of awareness of the risk of the disease and the benefits of vaccination and their attitudes. Discussions with parents about their sex life are positive, as adolescents are informed about how to protect themselves and the benefits of vaccination. The promotion of information actions, both for parents and their adolescent children, on the risk of this disease and its prevention through HPV vaccination, is necessary to reduce the fear and reluctance of parents towards vaccination.

## Appendices

Questionnaire concerning the knowledge of human papillomavirus infection and its prevention

Adolescents’ Identification Data

Adolescent’s initials: |__|__|__|

Age: |__|__|

Sex: __ M __ F

Educational qualification of the mother:

__ Middle school diploma __ High school diploma __ Degree

Educational qualification of the father:

__ Middle school diploma __ High school diploma __ Degree

Occupation of the mother:

__ Unemployed __ Housewife __ Industrial Worker __ Office Employee __ Manager __ Freelancer __ Other________________________

Occupation of the father:

__ Unemployed __ Industrial worker __ Office employee __ Manager __ Freelancer __ Other________________________

Type of occupation of the mother:

__ Full-time __ Part-time

Type of occupation of the father:

__ Full-time __ Part-time

Number of siblings:  F |__|__|   M |__|__|

Age of siblings: <10 years, no. |__|__|  10-14, no. |__|__| 14-18 years, no. |__|__| >18, no. |__|__| Questionnaire

Please, circle only one alternative for each question Knowledge of HPV infection Q1. Have you ever heard about HPV? 1. Yes 2. No 3. Do not remember Q2. Do you think that HPV could be dangerous? 1. Do not know 2. Yes 3. No 4. Only in subjects with chronic diseases Q3. How is HPV infection transmitted? 1. Do not know 2. Sexually 3. With kisses 4. With foods 5. Other, specify ________________ Q4. Have you ever heard of HPV vaccination? 1. Do not remember 2. No 3. Yes Q5. If yes, which is the main aim of HPV vaccination? 1. Do not know 2. Prevention of cervical cancer 3. Prevention of pregnancy 4. Prevention of a sexually transmitted disease 5. Other, specify_________________________________________ Q6. If yes, from which source? 1. Paediatrician 2. Another physician 3. Parents 4. Teachers 5. Friends 6. TV/radio 7. Internet 8. Other, specify _________________________________ Knowledge and personal attitudes towards HPV vaccination Q7. Do you think HPV infection might concern you? 1. Do not know 2. Yes 3. No Q8. Do you want to perform HPV vaccination? 1. Do not know 2. No 3. Yes Q9. If you do not want to perform HPV vaccination, why do you give this answer? 1. I do not know what it is aimed at 2. I am a male, and only females should be vaccinated 3. Fear of executing an injection 4. No fear of the illness related to HPV 5. Fear of vaccine- related adverse events 6. Religious reasons 7. Other, explain______________________________________________ Q10. If you want to perform HPV vaccination, why do you give this answer? 1. Prevention of a sexually transmitted disease 2. Prevention of a potentially carcinogenic infection 3. Other, specify________________________________________ Q11. Where would you like to be vaccinated against HPV? 1. Paediatrician 2. Gynecologist 3. Another physician 4. Local health unit 5. School 6. Hospital Q12. When is HPV vaccination recommended? 1. Do not know 2. Within the first year of life 3. Before the beginning of sexual activity 4. After the beginning of sexual activity 5. When a pregnancy is planned 6. Other, specify ____________________________________________ Q13. At what age do you think that information should be given about the possibility of HPV prevention? 1. Do not know 2. 10-13 years 3. 14-18 years 4. 18-30 years 5. >30 years Q14. Do you require more information on HPV and its prevention? 1. Do not know 2. No 3. Yes Q15. If you do not think that you require more information on HPV and its prevention, why do you give this answer? 1. HPV and its prevention do not interest me 2. Never had sexual intercourses 3. I always use condoms 4. This topic is not relevant 5. Other, specify_____________________________________________ Q16. If you require more information on HPV and its prevention, who should provide you with this information? 1. Paediatrician or general medicine doctor 2. Teacher at school 3. Other, specify_______________________________________ Sexual activity and attitudes towards discussing problems related to sexuality Q17. Do you have a boy/girl friend? 1. Yes 2. No Q18. Did you have at least once sexual intercourse? 1. Yes 2. No Q19. If yes, at which age did you have the first one? Specificy __________ years Q20. If yes, with how many persons have you had sexual intercourse? 1. One 2. Two 3. Three 4. More than three Q21.Do you use contraceptive methods? 1. No 2. Yes Q22. If yes, which ones? 1. Condom 2. Pill 3. Other, specify_________________ Q23. Who do you talk to about questions related to sexuality? 1. Friends 2. Parents 3. Teachers 4. Religious 5. Paediatrician 6. Family physician 7. Gynecologist 8. Other, specify ________________________________________ Q24. Do you talk about questions related to sexuality with your parents? 1. Never 2. Only if they begin to speak 3. Only if I have specific problems 4. Always Q25. Do you talk about sexually transmitted diseases with your parents? 1. Never 2. Only if they begin to speak 3. Only if I have specific problems 4. Always

Parent’s Identification Data

Parent’s initials: |__|__|__|

Age: |__|__|

Sex:  M  F

Educational qualifications: 

Medium school High-level school Degree

Occupation? 

 Unemployed Housewife Blue-collar worker White-collar worker Manager Freelancer Other________________________

Type of occupation: 

 Full-time Part-time

Number of sons/daughters: |__|__|

Age of sons/daughters attending the school investigated:

|__|__| |__|__| |__|__|

Gender of offspring attending the school investigated: M, no.___________  F, no.__________________

Is there someone in your family with a history of cancer? No Yes Is there someone in your family with a history of uterine cancer? No Yes

Attitudes towards vaccination in general Are you in favour of vaccinations in general? No Yes If no, why?  They are not always useful Because of potential adverse effects They might cause problems It is better to have the disease Other, specify ____________________

Why do you vaccinate your sons/daughters? It is compulsory Suggestion of the paediatrician Suggestion of the family physician I gather information from newspapers/TV I gather information on the Internet Other, specify _____________________

Which recommendation is significant to you to decide on a vaccination for your sons/daughters? Ministry of Health Regional Body Paediatrician Other physician Relative/friend Teacher Religious authority Other, specify _____________________ Questionnaire

Please, circle only one alternative for each question. Knowledge of HPV and HPV-related diseases Q1. Have you ever heard about HPV? 1. Yes 2. No Q2. Do you think that HPV could be dangerous? 1. Do not know 2. Yes 3. No 4. Only in subjects with chronic diseases Q3. How is HPV infection transmitted? 1. Do not know 2. Sexually 3. With strict contact 4. With foods 5. Other, specify ________________ Q4. What is the main aim of HPV vaccination? 1. Do not know 2. Prevention of cervical cancer 3. Prevention of pregnancy 4. Prevention of a sexually transmitted disease 5. Other, specify_________________________________________ Q5. Do you think that HPV could involve your sons/daughters? 1. Do not know 2. Yes 3. No Q6. If no, why? 1. They have no sexual intercourse yet. 2. They do not have sexual intercourse at risk. 3. They have no family history of cancer, 4. Other, specify_____________________________________________ Knowledge and personal attitudes towards HPV vaccination Q7. Do you want your sons/daughters to receive the HPV vaccination? 1. No 2. Yes 3. Maybe / not sure Q8. If you want your sons and/or daughters to receive the HPV vaccination, why do you give this answer? 1. Prevention of a sexually transmitted disease 2. Prevention of a potentially carcinogenic infection 3. Other, specify________________________________________ Q9. If you do not want your sons and daughters perform HPV vaccination, why do you give this answer? 1. Fear of executing an injection for my son/daughter 2. No fear of the illness 3. Fear of vaccine- related adverse events 4. Refusal of a vaccination that prevents a sexually transmitted illness 5. Religious reasons 6. Fear that the vaccination may encourage sexual activity 7. Other, explain______________________________________________ Q10. If you want your sons and/or daughters to perform HPV vaccination, what is the ideal age for giving them the vaccine? 1. Do not know 2. 10-11 years 3. 12-13 years 4. 14-18 years 5. >18 years Q11. When is HPV vaccination recommended? 1. Do not know 2. Within the first year of life 3. Before the beginning of sexual activity 4. After the beginning of sexual activity 5. When a pregnancy is planned 6. Other, explain ____________________________________________ Q12. Do you require more information on HPV and its prevention? 1. No 2. Yes Q13. If you do not think to require more information on HPV and its prevention, why do you give this answer? 1. HPV and its prevention do not interest me and my children 2. This topic is not relevant 3. Other, specify_____________________________________________ Q14. If you require more information on HPV and its prevention, which is the best way to increase your knowledge? 1. Simple and clear books in my language on this argument 2. Specific discussions with physicians of different specialities 3. Other, explain_____________________________________________ Q15. At what age do you think that the information about the possibility of HPV prevention? 1. I do not know 2. 10-11 years 3. 12-13 years 4. 14-18 years 5. >18 years Attitudes towards discussing problems related to sexuality with sons/daughters Q16. Do you talk about questions related to sexuality with your sons/daughters? 1. Never 2. Only if they begin to speak 3. Only if they have specific problems 4. Always Q17. Do you talk about sexually transmitted diseases with your sons/daughters? 1. Never 2. Only if they begin to speak 3. Only if they have specific problems 4. Always Q18.Do you think that your sons/daughters have to be informed about HPV and its prevention? 1. Yes 2. No Q19. If you think that your sons/daughters should not be informed about HPV and its prevention, why do you give this answer? 1. This topic is not interesting for them 2. Parents have to decide for their children 3. Religious reasons 4. Other, specify_________________________________________ Q20. If you think that your sons/daughters have to be informed about HPV and its prevention, who should give them the information? 1. Paediatrician / Family physician 2. Teacher at school 3. Other, specify_______________________________________

## References

[1] Tota JE, Bentley J, Blake J (2017). Introduction of molecular HPV testing as the primary technology in cervical cancer screening: acting on evidence to change the current paradigm. Prev Med.

[2] Sakamoto J, Shigehara K, Nakashima K (2019). Etiological role of human papillomavirus infection in the development of penile cancer. Int J Infect Dis.

[3] Tsikouras P, Paraskevopoulos K, Bothou A (2020). Tongue cancer and pregnancy: report of two cases in association with HPV infection and literature review. Is HPV vaccination necessary to prevent tongue cancer?. J Syst Integr Neurosci.

[4] Tristram A, Fiander A (2007). Human papillomavirus (including vaccines). Obstet Gynaecol Reprod Med.

[5] Burd EM (2003). Human papillomavirus and cervical cancer. Clin Microbiol Rev.

[6] Cheng L, Wang Y, Du J (2020). Human papillomavirus vaccines: an updated review. Vaccines (Basel).

[7] Harper DM, DeMars LR (2017). HPV vaccines - a review of the first decade. Gynecol Oncol.

[8] Pelucchi C, Esposito S, Galeone C (2010). Knowledge of human papillomavirus infection and its prevention among adolescents and parents in the greater Milan area, Northern Italy. BMC Public Health.

[9] Smolarczyk K, Duszewska A, Drozd S, Majewski S (2022). Parents' knowledge and attitude towards HPV and HPV vaccination in Poland. Vaccines (Basel).

[10] Grandahl M, Paek SC, Grisurapong S, Sherer P, Tydén T, Lundberg P (2018). Parents' knowledge, beliefs, and acceptance of the HPV vaccination in relation to their socio-demographics and religious beliefs: a cross-sectional study in Thailand. PLoS One.

[11] Rancic NK, Miljkovic PM, Deljanin ZM (2022). Knowledge about HPV infection and the HPV vaccine among parents in Southeastern Serbia. Medicina (Kaunas).

[12] Szilagyi PG, Albertin CS, Gurfinkel D (2020). Prevalence and characteristics of HPV vaccine hesitancy among parents of adolescents across the US. Vaccine.

[13] Shato T, Humble S, Anandarajah A (2023). Influences of sociodemographic characteristics and parental HPV vaccination hesitancy on HPV vaccination coverage in five US states. Vaccine.

[14] Lee YM, Riesche L, Lee H, Shim K (2018). Parental HPV knowledge and perceptions of HPV vaccines among Korean American parents. Appl Nurs Res.

[15] Seven M, Güvenç G, Şahin E, Akyüz A (2015). Attitudes to HPV vaccination among parents of children aged 10 to 13 years. J Pediatr Adolesc Gynecol.

[16] Ogochukwu TN, Akabueze J, Ezeome IV, Aniebue UU, Oranu EO (2017). Vaccination against human papilloma virus in adolescent girls: Mothers’ knowledge, attitude, desire and practice in Nigeria. J Infect Prev Med.

[17] Carvalho AMCD, Araújo TME (2021). Factors associated with adolescent compliance with human papillomavirus vaccine: a cross-sectional study. Texto Contexto Enferm.

[18] Santos AC, Silva NN, Carneiro CM, Coura-Vital W, Lima AA (2020). Knowledge about cervical cancer and HPV immunization dropout rate among Brazilian adolescent girls and their guardians. BMC Public Health.

[19] Icardi G, Costantino C, Guido M (2020). Burden and prevention of HPV. Knowledge, practices and attitude assessment among pre-adolescents and their parents in Italy. Curr Pharm Des.

[20] González-Cano M, Garrido-Peña F, Gil-Garcia E, Lima-Serrano M, Cano-Caballero MD (2021). Sexual behaviour, human papillomavirus and its vaccine: a qualitative study of adolescents and parents in Andalusia. BMC Public Health.

[21] Soares Junior JM, de Oliveira HM, Luquetti CM (2022). Adolescents' knowledge of HPV and sexually transmitted infections at public high schools in São Paulo: a cross-sectional study. Clinics (Sao Paulo).

[22] Oliveira MS, Sorpreso IC, Zuchelo LT (2020). Knowledge and acceptability of HPV vaccine among HPV-vaccinated and unvaccinated adolescents at Western Amazon. Rev Assoc Med Bras (1992).

